# Information Theory: Deep Ideas, Wide Perspectives, and Various Applications

**DOI:** 10.3390/e23020232

**Published:** 2021-02-17

**Authors:** Irad Ben-Gal, Evgeny Kagan

**Affiliations:** 1 Department of Industrial Engineering, Tel-Aviv University, Tel-Aviv 6997801, Israel; bengal@tauex.tau.ac.il; 2 LAMBDA Laboratory, Tel-Aviv University, Ramat Aviv 6997801, Israel; 3 Department of Industrial Engineering, Ariel University, Ariel 40700, Israel

The history of information theory, as a mathematical principle for analyzing data transmission and information communication, was formalized in 1948 with the publication of Claude Shannon’s famous paper “A Mathematical Theory of Communication” [1]. At the heart of the new theory stood the definition of the entropy measure of a random variable, as the average inherent “uncertainty” in the variable’s possible outcomes. However, the origins of this theory were incubated approximately seventy years earlier, when Willard Gibbs [2] and Ludwig Boltzmann [3] published their definition of entropy with a similar form and meaning to Shannon’s entropy. Gibbs and Boltzmann considered entropy within the framework of thermodynamics and used it as a characterization measure of the order in a mechanical system. They framed the laws of thermodynamics using the statistical properties of ensembles of possible states of a physical system composed of many particles.

In contrast to Boltzmann’s entropy, which relies on the number of system states, Gibbs’ and Shannon’s entropy relies on the probabilities of the system states while introducing the uncertainty concept into the entropy definition. This underling uncertainty also appears in the Von Neumann entropy, which is an extension of the classical Gibbs entropy concepts to the field of quantum mechanics.

Such an association between physical parameters and uncertainty led to a revolutionary change in intuitions underlying both physical research and philosophical speculations; even the contemporary cosmological theory is based on the entropy of black holes [4] and on the value which is considered as ‘Information of the Universe’ [5].

More surprising is a relation between entropy and modern mathematical theories, in which entropy appears both as an internal concept as well as an external parameter. For example, following the introduction of the entropy of partitions, and, consequently, the entropy of a map [6], it was applied to the analysis of dynamical systems and used to represent the structure of systems’ evolution. In later studies, the concept of entropy was introduced to algebraic geometry and used to characterize some properties of functional spaces [7]. Similarly, considerations of the entropy within the framework of combinatorics led to the development of algebraic information [8], which associates uncertainty in the sense of Shannon’s measure with the formal structure of combinatorial objects.

The relation between the entropy and uncertainty was further emphasized in optimal search and decision-making, where it was found that an optimal search procedure [9] is related to an optimal coding tree [10], while both are governed by similar search and optimization rules. In another example, studies in biophysics demonstrated that Gibbs entropy and corresponding statistical physics terms can well explain basic learning processes in neural networks [11]. Similarly, recent studies support the use of entropy and information to better understand the way that deep neural networks operate [12].

In the introduction to the well-known textbook “Elements of Information Theory” [13], Cover and Thomas discuss the relationship of the information theory with seven other fields with some leading applications, including mathematics (inequalities), computer science (Kolmogorov complexity), physics (AEP, thermodynamics, quantum information theory), communication theory (limits of communication), probability theory (limit theorems, large deviations), statistics (hypothesis testing, Fisher information), and economics (portfolio theory, Kelly gambling). Today, thirty years after the initial publication of this textbook and seventy years after Shannon’s publication, such a list can be complemented with many more applications in various levels and fields. These include data mining, search theory, machine learning, AI, optimization, robotics, control theory, game theory, decision making, industrial engineering, and biology, as well as even more distant research areas, such as neuroscience, philosophy, social sciences, and cognitive sciences. The wide variety of fields that are using concepts of information theory are represented in the different volumes of the Entropy journal and partially by this Special Issue, which focuses on the applications of information theory in industrial and service systems.

The current issue includes eight papers that represent various information theory applications in different fields of engineering and the industry. The paper titled Project Management Monitoring Based on Expected Duration Entropy by S. Cohen Kaspi, S. Rozenes, and I. Ben-Gal deals with classical industrial and management engineering problems by proposing an analytical method for identifying optimal inspection points in a project management using information theory measures. The paper titled Ordinal Decision-Tree-Based Ensemble Approaches: The Case of Controlling the Daily Local Growth Rate of the COVID-19 Epidemic by G. Singer and M. Marudi presents an ordinal decision tree approach in which an objective-based information gain measure is used to select the classifying attributes. The selected case study is extremely relevant to this epoch as it classifies areas with different growth rates of COVID-19. In contrast, the paper titled Event-Triggered Adaptive Fault Tolerant Control for a Class of Uncertain Nonlinear Systems by C. Zhu, C. Li, X. Chen, K. Zhang, X. Xin, and H. Wei solves the problem of adaptive fault-tolerant control in non-linear systems with uncertain feedback.

Other types of problems are considered in the paper titled Cooperative Detection of Multiple Targets by the Group of Mobile Agents by B. Matzliach, I. Ben-Gal, and E. Kagan and the paper titled Multi-Harmonic Source Localization Based on Sparse Component Analysis and Minimum Conditional Entropy by Y. Du, H. Yang, and X. Ma. The first paper presents information-theoretic based algorithms for the search and detection of hidden targets by a group of autonomous mobile agents, while the second paper applies sparse component analysis and minimum conditional entropy techniques and proposes a method for identifying multiple harmonic source locations in a distributed system.

New directions for using information theory measures are presented in two papers which consider the relation between entropy and other measures of uncertainty developed in fuzzy logic and apply new concepts for the analysis of industrial engineering problems. In particular, the paper titled Entropy-Based GLDS Method for Social Capital Selection of a PPP Project with q-Rung Orthopair Fuzzy Information by L. Liu, J. Wu, G. Wei, C. Wei, J. Wang, and Y. Wei considers the problem of social capital selection in a public-private partnership, for which the method of gained and lost dominance score with the q-rung orthopair fuzzy entropy is applied. The second paper, titled Negation of Pythagorean Fuzzy Number Based on a New Uncertainty Measure Applied in a Service Supplier Selection System, by H. Mao and R. Cai follows more traditional techniques of Pythagorean fuzzy numbers, but, in contrast to conventional studies, it implements a new definition of the entropy of Pythagorean fuzzy numbers and applies it for the evaluation of service quality in service systems with supplier selection.

Finally, the paper titled Inferring Authors’ Relative Contributions to Publications from the Order of their Names when Default Order is Alphabetical by Y. Gerchak demonstrates the effectiveness of the entropy measure to allocate the contribution of authors by the ordering of their names. This is a field that is traditionally considered to be strongly dependent on human judgment or game theory concepts. Such an application sheds a light on new perspectives of information-based decision-making and recommendation systems.

The variety of themes and applications considered even in this limited issue emphasize, once again, that the significant contributions and broadness of information theory concepts are not limited to the analysis of traditional communication systems. The use of these concepts allows us to explain a wide range of natural and artificial phenomena as well as to implement theoretical ideas and precise measures of uncertainty and information in engineering projects and complex systems.

In his famous lecture [14], Eugene Wigner discussed the unreasonable effectiveness of mathematics in natural sciences and, in particular, said “philosophy is the misuse of a terminology which was invented just for this purpose. In the same vein, I would say that mathematics is the science of skillful operations with concepts and rules invented just for this purpose”.

The concepts used in information theory are probabilities and bits; the first are continuous measures of uncertainty, while the second are discrete levels of truth. Today, it seems meaningful to follow Wigner’s thinking and discuss the immense effectiveness of information theory concepts in human reasoning and complex systems’ operation and analysis.
